# A Chemo-Enzymatic Road Map to the Synthesis of CoA Esters

**DOI:** 10.3390/molecules21040517

**Published:** 2016-04-20

**Authors:** Dominik M. Peter, Bastian Vögeli, Niña Socorro Cortina, Tobias J. Erb

**Affiliations:** 1 Institute for Microbiology, Eidgenössische Technische Hochschule (ETH) Zürich, Vladimir-Prelog-Weg 4, CH-8093 Zürich, Switzerland; peterdom@ehtz.ch; 2 Biochemistry & Synthetic Biology of Microbial Metabolism Group, Max-Planck-Institute for Terrestrial Microbiology, Karl-von-Frisch-Str. 10, D-35043 Marburg, Germany; bastian.voegeli@mpi-marburg.mpg.de (B.V.); nina.cortina@mpi-marburg.mpg.de (N.S.C.)

**Keywords:** CoA-thioester, acylation, acyl-CoA dehydrogenases, enzyme promiscuity, secondary metabolites, polyketide synthase, natural product biosynthesis, biocatalysis, extender units

## Abstract

Coenzyme A (CoA) is a ubiquitous cofactor present in every known organism. The thioesters of CoA are core intermediates in many metabolic processes, such as the citric acid cycle, fatty acid biosynthesis and secondary metabolism, including polyketide biosynthesis. Synthesis of CoA-thioesters is vital for the study of CoA-dependent enzymes and pathways, but also as standards for metabolomics studies. In this work we systematically tested five chemo-enzymatic methods for the synthesis of the three most abundant acyl-CoA thioester classes in biology; saturated acyl-CoAs, α,β-unsaturated acyl-CoAs (*i.e.*, enoyl-CoA derivatives), and α-carboxylated acyl-CoAs (*i.e.*, malonyl-CoA derivatives). Additionally we report on the substrate promiscuity of three newly described acyl-CoA dehydrogenases that allow the simple conversion of acyl-CoAs into enoyl-CoAs. With these five methods, we synthesized 26 different CoA-thioesters with a yield of 40% or higher. The CoA esters produced range from short- to long-chain, include branched and α,β-unsaturated representatives as well as other functional groups. Based on our results we provide a general guideline to the optimal synthesis method of a given CoA-thioester in respect to its functional group(s) and the commercial availability of the precursor molecule. The proposed synthetic routes can be performed in small scale and do not require special chemical equipment, making them convenient also for biological laboratories.

## 1. Introduction

Coenzyme A (CoA) plays a vital role in all three domains of life. Its thioesters are key intermediates in many metabolic and biosynthetic pathways. Approximately 5% of all enzymes listed on the BRENDA database use CoA as a substrate or produce it [1]. This includes important enzymes in primary metabolism (e.g., acetyl-CoA carboxylase, fatty acid synthase, pyruvate dehydrogenase, *etc.*), as well as enzymes involved in natural product biosynthesis (e.g., polyketide synthases). Access to CoA thioesters is crucial for studying and engineering the biochemistry of these enzymes and pathways [2]. Commercial availability of CoA esters, however, is limited to only a few compounds, such as acetyl-CoA or malonyl-CoA. This fact often requires researchers to synthesize their own CoA-thioesters starting from CoA or the even more basic building block pantothenic acid [3]. The main difficulties in working with CoA are its insolubility in organic solvents due to the three phosphate groups in the CoA backbone, as well as the presence of several other functional groups that interfere with the use of many chemical reactants. A number of different synthesis routes for the acylation of CoA are available in the literature [2,4,5,6]. Unfortunately, these routes are very often only specific descriptions for one particular compound. Moreover, the synthetic protocols are of very chemical nature and require some synthetic chemistry, for which many biology laboratories are not well equipped. To sort the plethora of synthetic protocols for the most efficient and convenient methods, we systematically tested different synthetic routes for 30 different compounds. This allowed us to provide a roadmap for the convenient synthesis of a large variety of biologically relevant CoA-thioester based on functional groups.

## 2. Results

**Figure 1:**
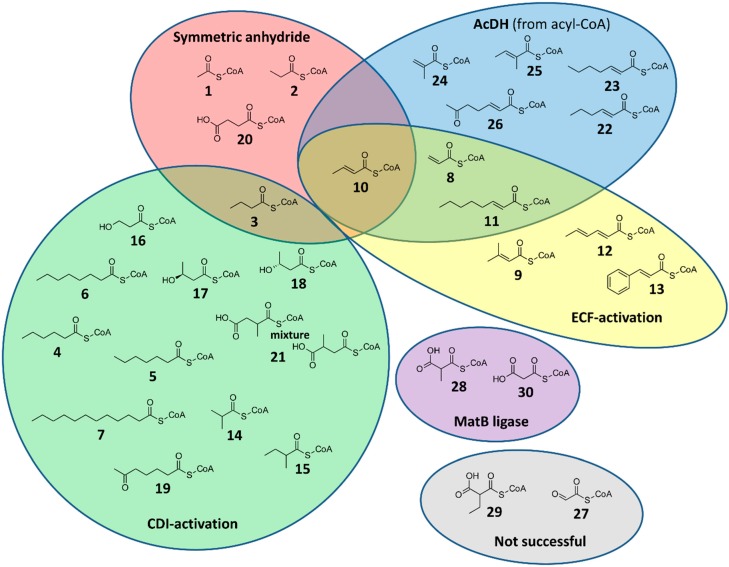
Acyl-CoA thioesters that were successfully synthesized by one of the five different methods tested in this study. Color code: symmetric anhydride method, red; CDI-activation, green; ECF-activation, yellow; acyl-CoA-desaturation by AcDHs, blue; ATP dependent ligation by MatB, purple. Note that not all methods were tested with every substrate.

We systematically tested three different chemical CoA acylation methods for their applicability on 26 model compounds and screened three acyl-CoA dehydrogenases for their synthetic use on 12 different CoA-thioesters (Figure 1). We also checked the previously described CoA-ligase MatB for the enzymatic synthesis of the biologically highly relevant malonyl-CoA (**30**) and methylmalonyl-CoA (**28**) and determined the stereochemical outcome of the latter [7]. We analyzed all reactions by analytical HPLC coupled to a UV-Vis detector and a mass spectrometer (Figure S1). To determine the yield of chemical reactions, as well as MatB catalyzed syntheses, we measured the absorbance at 260 nm and calculated the ratio of product peak area to total peak area (Table 1). For the acyl-CoA dehydrogenase screen we calculated conversion rate by the ratio of substrate to product peak area. The individual methods tested and their limitations are described in the following.

### 2.1. CoA Acylations by Symmetric Anhydride

Arguably the most convenient and cleanest method of CoA-acylation we tested was the direct thioesterification of an acyl substrate in the form of a symmetric anhydride with free CoA. The reaction yielded almost no side products and could be done in buffered water. The method worked reliably for the following compounds with overall yields of more than 80%: Acetyl-CoA (**1**), propionyl-CoA (**2**), butyryl-CoA (**3**), crotonyl-CoA (**10**), and succinyl-CoA (**20**) (Table 1). Unfortunately, the number of commercially available symmetric anhydrides is limited to short, unbranched alkanes and alkenes chains, which limited application of this method to the synthesis of other, more complex CoA-thioesters.

### 2.2. CoA Acylations by Carbonyldiimidazole (CDI)

The second CoA-acylation method we tested was a two-step protocol, in which free carboxylic acids are activated *in situ* with CDI, followed by the direct thioesterification of the CDI-activated intermediate with free CoA [5]. Because every carboxylic acid commercially available is a potential starting material for this method, the pool of potential acyl-CoAs tested was significantly larger than that for the symmetric anhydride method.

In total, we screened 21 different carboxylic acids for CDI-mediated CoA-acylation. 11 out of the 21 measured syntheses showed a yield of 50% or more (Table 1). In particular the synthesis of aliphatic acyl-CoAs worked reliably, the only exception being butyryl-CoA (**3**) with a yield of only 40% (more than a 20% difference from other aliphatic acyl-CoAs). We also successfully coupled carboxylic acids that carry functional groups to CoA using CDI as activating agent. These functionalized acids included primary alcohols: 3-hydroxypropionyl-CoA (**16**) 66% yield, secondary alcohols: (*R*)- and (*S*)-3-hydroxybutyryl-CoA **17** and **18**, in 54% and 57% yield, respectively, as well as ketones: 6-oxoheptanoyl-CoA (**19**), 56% yield. Notably, the method was not susceptible to considerable quantities of water. For the synthesis of **16**, the 3-hydroxypropanoic acid used as starting material was a 30% solution in H_2_O (to prevent spontaneous polyesterification), yet the yield, 66%, seemed unaffected compared to other, water-free syntheses (e.g., **17** and **18** with 54% and 57% yield).

CDI-mediated CoA-acylation of dicarboxylic acids was less successful. Only methylsuccinyl-CoA (**21**) could be synthesized with a yield of 40%. The reaction product was most likely a mixture of the 2- and 3-methyl isomers, which were not separable using our HPLC analysis conditions. In the cases of ethylmalonic and methlymalonic acid we detected large amounts of the corresponding decarboxylated acyl-CoAs: butyryl-CoA (**3**, 40%) and propionyl-CoA (**2**, 41%), respectively. It is unclear whether the decarboxylation took place after activation by CDI or after the compounds were coupled to CoA. We were also unable to reliably produce α,β-unsaturated acyl-CoAs (enoyl-CoAs) using CDI-activation. This was probably due to π-conjugation of the α,β double bond with the carboxylic acid, which lowers the reactivity of the precursor compounds. The syntheses with α,β-unsaturated carboxylic acids as educts generally showed yields below 10%: sorbityl-CoA (**12**, 9%), 3,3-dimethylacrylyl-CoA (**9**, 4%), octenoyl-CoA (**11**, 2%), cinnamoyl-CoA (**13**, 1%) and crotonyl-CoA (**10**, <1%).

### 2.3. CoA Acylations by Ethylchloroformate (ECF)

As an alternative to the CDI-method, we tested ECF, which forms a more reactive intermediate compound, as activating agent. Overall, the average yield for successful CoA-acylation reactions with aliphatic acids was slightly lower with ECF-mediated couplings (49% yield) than for CDI-mediated couplings (62% yield). This is due to the fact that ECF-activated carboxylic acids form a mixed anhydride. The free CoA can attack on two different positions, resulting in either the desired product or propoxycarbonyl-CoA as side product. Thus, for aliphatic acids as starting material, the CDI-method is the preferred choice compared to the ECF-method.

For the synthesis of biologically highly relevant α,β-unsaturated acyl-CoA thioesters, which were not accessible through CDI-activation, ECF-mediated coupling was well suited. We successfully synthesized: acrylyl-CoA (**8**) 17% yield, crotonyl-CoA (**10**) 44% yield, octenoyl-CoA (**11**) 57% yield, sorbityl-CoA (**12**) 61% yield 3,3-dimethylacrylyl-CoA (**9**) 39% yield, and cinnamoyl-CoA (**13**) 75% yield (Table 1). We did not detect any methylmalonyl-CoA (**28**), ethylmalonyl-CoA (**29**) or glyoxylyl-CoA (**27**). ECF-coupling is thus the preferred chemical synthesis method for enoyl-CoA compounds.

### 2.4. Desaturation of Acyl-CoAs

**Figure 2:**
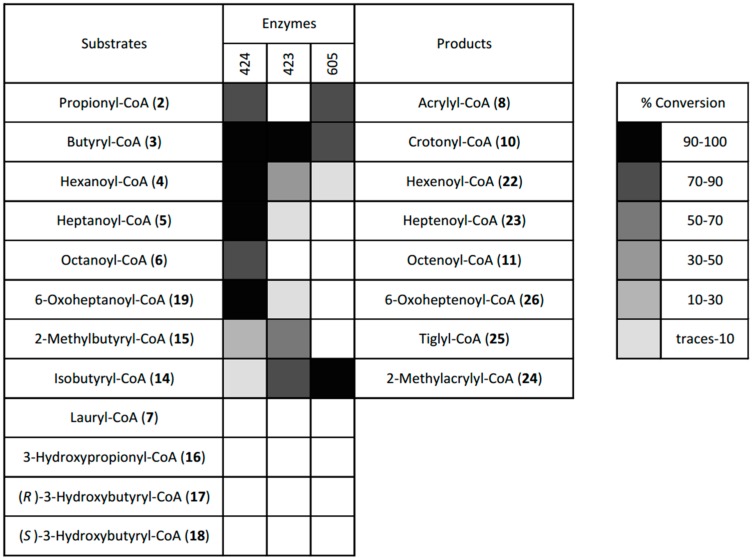
Substrate promiscuity of the three acyl-CoA dehydrogenases characterized in this study. The following enzymes were cloned from *Rhodobacter sphaeroides* and produced heterologously in *Escherichia coli*: medium-chain acyl-CoA dehydrogenase, 424; putative short-chain acyl-CoA dehydrogenase, 423; putative branched-chain acyl-CoA dehydrogenase, 605. Conversion rates were measured by HPLC-UV at 260 nm as described in material and methods.

As an alternative route to synthesize α,β-unsaturated acyl-CoAs we tested the possibility to combine CDI–mediated synthesis of acyl-CoA thioesters with a subsequent enzymatic desaturation step. In previous work, we had used a medium chain acyl-CoA dehydrogenase (AcDH) from *Rhodobacter sphaeroides* to synthesize the α,β-unsaturated acyl-CoA thioesters heptenoyl-CoA (**23**), octenoyl-CoA (**11**), nonenoyl-CoA and 6-oxoheptenoyl-CoA (**26**) from their corresponding saturated acyl-CoA precursors [8]. To more systematically identify and characterize the synthetic potential of acyl-CoA desaturating enzymes, we cloned and tested three AcDH members from different sub-branches of the large acyl-CoA dehydrogenase family [9]: the medium-chain AcDH (424) reported before [8], a putative short chain-CoA AcDH (423), as well as a putative branched chain-CoA AcDH (605). We screened all acyl-CoAs that we successfully produced with CDI-activation against these three different AcDHs. The progress of each reaction could be easily evaluated visually by following the color change from light blue to yellow during successful desaturation (see Figure S2). Exact conversion rates were determined by HPLC-UV-Vis analytics after 2 h of incubation with each enzyme (Figure S3). In line with the predicted function of each AcDH, the tested enzymes exhibited either promiscuity towards medium chain acyl-CoA derivatives (424) or were more restricted to butyryl-CoA (423) or isobutyryl-CoA (605) and closely related compounds (Figure 2). Together, the three AcDHs tested, covered a range of short-, medium- and branched-chain acyl-CoA substrates with overlapping promiscuities and yields between 50% and 100%. These include hexenoyl-CoA (**22**), heptenoyl-CoA (**23**), 2-methylacrylyl-CoA (**24**), tiglyl-CoA (**25**) and 6-oxoheptenoyl-CoA (**26**), all of which we could not produce with others methods. The only compounds that were not desaturated by any of the three enzymes were lauryl-CoA, 3-hydroxypropionyl-CoA and 3-hydroxybutyryl-CoA (the latter two compounds would have been anyways expected to tautomerize to the corresponding 3-oxo-acyl-CoA compounds). In summary, this chemo-enzymatic two-step process is a reliable method for the synthesis of enoyl-CoA esters that is especially valuable if the precursor acids are not commercially available for ECF-activation (e.g., heptenoic acid, octenoic acid, or noneoic acid). The method can also be used for *in situ* production of relatively reactive and instable enoyl-CoAs such as methylacrylyl-CoA (**24**) or acrylyl-CoA (**8**).

### 2.5. Enzymatic CoA Acylation by MatB

To synthesize the biologically highly relevant compound malonyl-CoA (**30**) 95% yield and methylmalonyl-CoA (**28**) 92% yield we used the previously described ligase MatB [7]. We determined the stereochemical outcome of the methylmalonyl-CoA synthesis enzymatically, adapted from a recently established method [10,11]. To that end, we incubated *in situ*-produced methylmalonyl-CoA with (*S*)-methylmalonyl-CoA mutase and succinyl-CoA reductase in combination with or without methylmalonyl-CoA racemase (Figure S3). This experiment confirmed that the (*R*)-stereoisomer of methylmalonyl-CoA is produced preferentially.

## 3. Discussion

**Figure 3:**
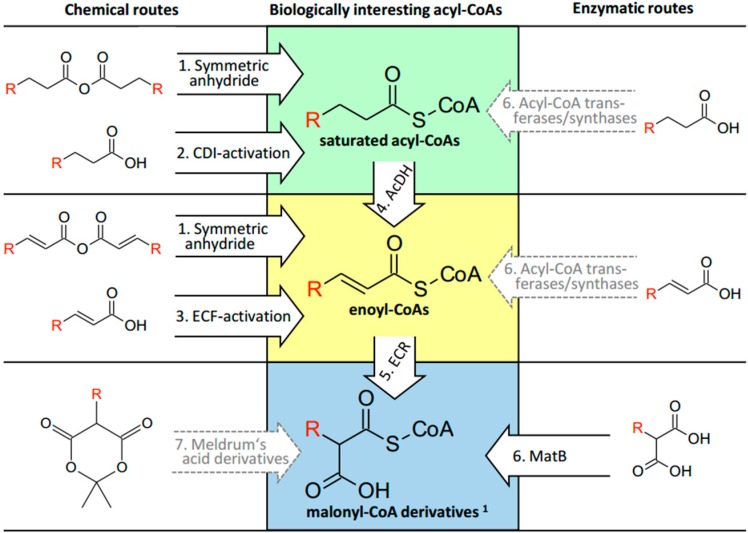
Road map for the synthesis of biologically relevant CoA-thioesters. Shown in bold arrows are the five routes that have been established by our group (**Routes 1**–**4**, this study; **Route 5** [8]; **Route 6**, this study and [12,13]). These routes give access to all three classes of acyl-CoA esters with high yield and stereoselectivity. Alternative routes that are also discussed in the text are shown with dashed arrows. Abbreviations: AcDH, acyl-CoA dehydrogenases; ECR, enoyl-CoA reductases/carboxylases; MatB, methylmalonyl-CoA synthase. ^1^ Note that ECR (**Route 5**) produces the biologically relevant (*S*)-malonyl-CoA stereoisomers, whereas matB (**Route 6**) yields the (*R*)-stereoisomers and Meldrum’s acid (**Route 7**) derivatives a racemic mixture thereof.

We successfully synthesized 28 different CoA-thioesters using five different methods, all of which can be carried out in standard plastic tubes used in most biology labs. We only failed to produce two of the targeted acyl-CoA esters: glyoxylyl-CoA (**27**) and ethylmalonyl-CoA (**29**). Judging by the yield and ease of use of our model syntheses we provide a roadmap of which method to use for each individual acyl-CoA class (Figure 3).

### 3.1. Saturated Acyl-CoA Esters

For saturated acyl-CoA esters the preferred method is the direct thioesterification of a symmetric anhydride with free CoA (**Route 1**). If the symmetric anhydride is not commercially available, we suggest CDI-mediated coupling (**Route 2**) as the second method of choice. It offers generally very high yields, is still very convenient and does not require the use of hazardous or expensive chemicals. We successfully synthesized acyl-CoAs with CDI-activation that contain the following functional groups: primary and secondary alcohols as well as ketones.

### 3.2. α,β-Unsaturated Enoyl-CoA Esters

As for saturated acyl-CoA esters, the thioesterification of a symmetric anhydride with free CoA is also the most convenient synthetic way for α,β-unsaturated enoyl-CoA esters (**Route 1**). The small selection of commercially available symmetric anhydrides limits this route. CDI-activation, however, was not suited for α,β-unsaturated enoyl-CoA esters. Therefore we propose two additional routes. The first route is the ECF-mediated chemical coupling of the α,β-unsaturated precursor acid with CoA (**Route 3**). This method requires more aggressive chemicals than CDI and might offer slightly lower yields, but worked very reliable for the synthesis of all α,β-unsaturated acyl-CoAs tested. As an alternative route we suggest CDI-mediated coupling of the saturated precursor acid (analogous to **Route 1**) followed by enzymatic desaturation of the produced acyl-CoA to the desired α,β-unsaturated enoyl-CoA ester (**Route 4**), for which we characterized three new enzymes in this study. One major advantage of this alternative route is the larger availability of saturated precursor acids compared to that of α,β-unsaturated precursor acids needed for ECF-coupling (this holds especially true for ^13^C/^2^H isotopically labeled acids). Enzymatic desaturation can be applied to lyophilized crude CDI-synthesis without the necessity of an intermediary purification step, which adds to the convenience of this two-step method. Additionally, this method offers the possibility to generate reactive desaturated acyl-CoAs (e.g., acrylyl-CoA (**8**)) in reaction mixtures *in situ*. As an alternative enzymatic route many medium- and long-chain saturated and α,β-unsaturated acyl-CoAs can be produced using the promiscuous fatty acid CoA ligases from *Mycobacterium tuberculosis* [14] (**Route 6**).

### 3.3. α-Carboxylated Malonyl-CoA Derivatives

For the synthesis of malonyl-CoA derivatives that serve as extender units in polyketide biosynthesis and are therefore of special interest, we recently described an efficient enzymatic synthetic route [8]. This method allowed us to produce a library of 18 different α-carboxylated (*S*)-malonyl-CoA derivatives from the corresponding α,β-unsaturated enoyl-CoA thioester through the reductive carboxylation by a promiscuous, engineered enoyl-CoA carboxylases/reductases (ECR, **Route 5**, [8,15]). To produce the enoyl-CoA precursor for the ECR reaction, we propose to use ECF-activation (**Route 3**). Alternatively, the α,β-unsaturated enoyl-CoA precursor can be generated enzymatically from their acyl-CoA derivatives via the one of the three AcDHs, characterized in this study (**Route 4**). Note, that both synthetic routes require the lyophillization or purification of the corresponding enoyl-CoA precursor prior to reductive carboxylation (**Route 5**).

For the common compounds malonyl-CoA and methylmalonyl-CoA (**28**) the recently described methylmalonyl-CoA ligase MatB from *Rhizobium trifolii* is a reliable direct enzymatic synthesis route from free (methyl-)malonic acid (**Route 6**, [7,16]). Through active site mutagenesis, a more promiscuous MatB variant was created recently. This variant was used to generate other α-carboxylated (*R*)-malonyl-CoA derivatives *in situ* for *in vitro* polyketide synthesis assays [12,16]. Note however, that MatB synthesis is limited by commercial availablility of free malonic acid starting precursors. Moreover, reductive carboxylation through ECR as described above directly produces the (*S*)-stereoisomer [17], which is used in polyketide biosynthesis. In contrast, MatB yields the (*R*)-stereoisomer, which requires an epimerase or racemization reaction to efficiently generate the biosynthetically more relevant (*S*)-stereoisomer. For certain biochemical applications (e.g., polyketide biosynthesis), the spontaneous racemization at the acidic α-position of malonyl-CoA derivatives might be sufficient. If a certain α-carboxylated malonyl-CoA derivative would not be accessible through either enzymatic route (**Route 5** or **6**), it could be chemically synthesized in multiple reaction steps via ring opening of a Meldrum’s acid derivative [18] as a last alternative (**Route 7**).

In conclusion, our experiences described above will serve as guideline for the synthesis of a given CoA thioester in analytical, semi-preparative and preparative scale from simple chemicals and with minimal equipment. The enzymes required (the three newly characterized AcDHs and the reported promiscuous ECR [8]) will be made available to the community through the Addgene plasmid repository.

## 4. Materials and Methods

### 4.1. Symmetric Anhydride Synthesis

For the small scale synthesis of butyryl-CoA and succinyl-CoA with the symetric anhydride method, CoA (4 mg, 0.005 mmol, 1 eq.) was dissolved in 200 µL 0.5 M NaHCO_3_. The solution was cooled down to 4 °C and 1.6 equivalents of the corresponding anhydride was added (butyryic anhydride: 1.3 µL, 0.0081 mmol, 1.6 eq.; succinic anhydride: 0.81 mg, 0.0081 mmol, 1.6 eq.). The reaction was stirred on ice for 45 min and its completion confirmed by a test for remaining free thiols with Ellman’s reagent. The reaction mixture was directly injected into the HPLC-MS for downstream analysis (see Section 4.6).

The synthesis of acetyl-CoA, propionyl-CoA and crotonyl-CoA was scaled up 50 fold using 200 mg CoA (0.25 mmol, 1 eq.) in 5 mL 0.5 M NaHCO_3_ and 1.6 eq. of the corresponding anhydride (acetic anhydride: 45 µL, 0.41 mmol, 54 µL 1.6 eq. propionic anhydride: 0.41 mmol, 1.6 eq.; crotonic anhydride: 64 µL 0.41 mmol, 1.6 eq.). The reactions were stirred on ice for 45 min and then directly injected into the HPLC-MS for downstream analysis and purification (see Section 4.6).

### 4.2. Carbonyldiimidazole Synthesis

For the carbonyldiimidazole (CDI) synthesis screen of acyl-CoA 4.2 mg CDI (0.026 mmol, 4 eq.) was dissolved in 200 µL THF, the corresponding acid was added (0.031 mmol, 4.8 eq.) and the mixture stirred at 22 °C for 1 h. 5 mg CoA (0.0064 mmol, 1 eq.) was dissolved in 50 µL of 0.5 M NaHCO_3_ and added to the reaction mixture. The reactions were stirred for another 45 min at 22 °C, flash frozen in liquid N_2_ and lyophilized overnight. The samples were then dissolved in 600 µL H_2_O and used for analysis by HPLC-MS (see Section 4.6) as well as for the dehydrogenase screen (see Section 4.4.3).

### 4.3. Ethylchloroformate Synthesis

For the small scale synthesis of acyl-CoAs with the ethylchloroformate synthesis the corresponding acid (0.051 mmol, 10 eq.) was dissolved in 200 µL THF, cooled to 4 °C, 3.6 µL triethylamine (0.026 mmol, 5 eq.) and 2.6 µL ethylchloroformate (0.026 mmol, 5 eq.) was added and the mixture was stirred for 45 min at 4 °C. 4 mg CoA (0.0051 mmol, 1 eq.) was dissolved in 200 µL of 0.5 M NaHCO_3_ and added to the reaction mixture. The reactions were stirred for another 45 min at 22 °C, flash frozen in liquid N_2_ and lyophilized overnight. The samples were then dissolved in 480 µL H_2_O and used for analysis by HPLC-MS (see Section 4.6) as well as for the dehydrogenase screen (see Section 4.4.3).

### 4.4. Dehydrogenase Screen

#### 4.4.1. Cloning

The genes of the two cloned dehydrogenases from *Rhodobacter sphaeroides* were amplified from chromosomal DNA using Phusion polymerase (Fermentas, Zug, Switzerland) in HF buffer; the PCR cycles consisted of initial denaturation for 3 min at 95 °C, followed by 30 cycles of 30 s denaturation at 95 °C, 20 s annealing at 55 °C, 50 s extension at 72 °C and a final extension step for 5 min at 72 °C. The butyryl-CoA dehydrogenase YP_354698 gene from *Rhodobacter sphaeroides* was amplified from chromosomal DNA by PCR using the forward primer GGATTTCATATGAGCGTCCTGACCGAC and the reverse primer GCATTTGGATCCTCACGCCA TGCCCCTGAG introducing a *NdeI* and *BamHI* cut site. The DNA fragment was digested with *NdeI* and *BamHI* and ligated into the expression vector pET28b generating the N-terminal His_6_ tagged expression construct pTE423. The isobutyryl-CoA dehydrogenase YP_353229 gene from *Rhodobacter sphaeroides* was amplified with the forward primer CGGATCGCATATGGATTTCGCGCTGAGCG AG and the reverse primer GTACATGAATTCTCATGCGGCCCCCAAGGC introducing a *NdeI* and an *EcoRI* restriction site. It was cloned into pET16b generating a N-terminal His_10_ tagged expression construct pTE605. The cloning of the expression construct pTE424 for the medium-chain dehydrogenase from *Streptomyces coelicolor* NP_627272.1 was previously described [8]. The protein sequences of the three dehydrogenases can be found in Table S2 and the corresponding DNA sequence in Table S3.

#### 4.4.2. Expression and Purification

*E. coli* BL21 (DE3) was transformed with the respective expression construct and a preculture was grown overnight at 30 °C in 5 mL LB containing 100 µg/mL Ampicillin for pTE605 or 50 µg/mL kanamycin for pTE423 and pTE424. The overnight culture was used to inoculate 500 mL of TB containing the respective antibiotic. The culture was grown to an OD_600_ of 0.8 at 37 °C, cooled down to 23 °C, induced with 0.25 mM IPTG and grown for another 12 h. Cells were harvested by centrifugation, resuspended in 10 mL Lysis Buffer (50 mM Tris-HCl pH 7.8, 500 mM NaCl, 10% glycerol, SIGMAFAST™ protease inhibitor) per gram cell pellet. All the proceeding steps were done on ice. The cells were lysed by sonication, the lysate was centrifuged for 60 min at 42,000× *g*. The supernatant was loaded onto a 1 mL Ni-Sepharose HisTrap FF column (GE Healthcare, Glattbrug, Switzerland) that had been equilibrated with Buffer A (50 mM Tris-HCl pH 7.8, 500 mM NaCl). The column was washed with 18% Buffer B (50 mM Tris-HCl pH 7.8, 500 mM NaCl, 500 mM imidazole) and eluted with 100% Buffer B. The purity was assessed by SDS-PAGE analysis (Figure S4). The enzyme concentration was determined by a Bradford assay.

#### 4.4.3. Screen of the Three Dehydrogenases

The lyophilized reaction mixtures from the carbonyldiimidazole synthesis were resuspended in 600 µL of H_2_O. Assuming a full conversion of the used CoA for the chemical synthesis, the resulting solutions contained 10 mM acyl-CoA. The screening assays were run at room temperature in 1 mL 100 mM NaHPO_4_ buffer at pH 8 containing 1 µM of the tested dehydrogenase, 10 µM FAD, 3 mM ferrocenium hexafluorophosphate and 1 mM of the resuspended acyl-CoA reaction mixture. The reaction was incubated for 2 h at room temperature and then quenched with 20 µL of 50% formic acid. The mixture was centrifuged at 17,000× *g* for 10 min and injected into the HPLC-UV-MS for analysis.

### 4.5. CoA Ligation by MatB and Determination of the Stereochemistry of Methylmalonyl-CoA

For the small scale synthesis of malonyl-CoA and methylmalonyl-CoA the CoA ligase MatB was used. CoA (5 mg, 0.0064 mmol, 1 eq.), malonic acid (3.3 mg, 0.032 mmol, 4 eq.) or methylmalonic acid (3.8 mg, 0.032 mmol, 4 eq.) and ATP (17.6 mg, 0.032 mmol, 4 eq.) was dissolved in 1.3 mL 200 mM NH_4_HCO_3_ pH 6.8 containing 15 mM MgCl_2_. The reactions were incubated at 30 °C and their completion confirmed by a test for remaining free thiols with Ellman’s reagent. The reaction mixture was directly injected into the HPLC-MS for downstream analysis (see Section 4.6). The stereochemical outcome of methylmalonyl-CoA produced by MatB it was coupled *in situ* to a specific (*S*)-methylmalonyl-CoA mutase (Mcm) [10] and succinyl-CoA reductase (SucD) [11]. The reaction was followed at 340 nm using an absorption coefficient for NADPH of ε_340 nm_ = 6.2 cm^−1^ mM^−1^. An assay of 100 µL 100 mM NaPO_4_ buffer at pH 8 contained 840 µM ATP, 520 µM CoA, 250 µM NADPH, 4 mM MgCl_2_, 20 mM KCl, 82 µg SucD, 16.5 µg Mcm, 90 µg MatB and was started by adding 2 µL of 5 mM methylmalonate (100 µM end concentration). After the reaction reached a steady state 1 µL of the methylmalonyl-CoA racemase (5 µg) was added.

### 4.6. Yield Determination

To measure the yield of the chemical coupling reactions with all three methods as well as for the dehydrogenase screen, 5 µL of the resuspended reaction mixtures diluted to 1 mM acyl-CoA was injected into an 1600 Infinity HPLC-UV system (Agilent Technoligies, Waldbronn, Germany) with a Quadrupol LC/MS detector 6130. The samples were separated over a Phenomenex 3 µ C18 column with a flow rate of 0.150 mL/min in 25 mM NH_4_HCO_2_ buffer pH 8.2. Dicarboxylic acids were eluted over 50 min with a methanol gradient from 1% to 45%; the short-chain acyl-CoAs with a gradient from 5% to 50% and the long-chain acyl-CoAs (starting with heptenoyl-CoA) with a gradient from 45% to 90%. To determine the product yield of the chemical synthesis the UV trace at 260 nm of the product peak (identified by MS) was integrated and given as a percentage of the total absorption at 260 nm (the characteristic injection peak was removed beforehand). For the dehydrogenase screen the total peak area of the saturated substrate and the unsaturated product was divided by their extinction coefficient at 260 nm (16.4 cm^−1^ mM^−1^ for saturated acyl-CoAs, 22.4 cm^−1^mM^−1^ for unsaturated enoyl-CoAs [19]) to correct for the difference in extinction. The conversion rate was calculated by dividing the normalized total peak area of the product by the normalized total peak area of the substrate.

## Figures and Tables

**Table 1:** Chemically synthesized CoA-thioesters. Products were confirmed by HPLC-MS. Yield was determined with HPLC-UV at 260 nm.

Acyl-CoA	Yield (%)	Used Method ^1^
Acetyl-CoA **1**	81	anhydride
Propionyl-CoA **2**	86	anhydride
Butyryl-CoA **3**	87	anhydride
Hexanoyl-CoA **4**	76	CDI
Heptanoyl-CoA **5**	74	CDI
Octanoyl-CoA **6**	68	CDI
Lauryl-CoA **7**	67	CDI
Acrylyl-CoA **8**	17	ECF
3,3-Dimethylacrylyl-CoA **9**	39	ECF
Crotonyl-CoA **10**	80	anhydride
Octenoyl-CoA **11**	57	ECF
Sorbityl-CoA **12**	61	ECF
Cinnamoyl-CoA **13**	75	ECF
Isobutyryl-CoA **14**	68	CDI
2-Methylbutyryl-CoA **15**	78	CDI
3-Hydroxypropionyl-CoA **16**	66	CDI
3-(*R*)-Hydroxybutyryl-CoA **17**	54	CDI
3-(*S*)-Hydroxybutyryl-CoA **18**	57	CDI
6-Oxoheptanoyl-CoA **19**	56	CDI
Succinyl-CoA **20**	86	anhydride
Methylsuccinyl-CoA **21**	40	CDI

^1^ If multiple synthesis methods were successful only the one with the highest yield is listed (see also Table S1).

## Supplementary Materials

Supplementary materials can be accessed at: http://www.mdpi.com/1420-3049/21/4/517/s1.

## Abbreviations

The following abbreviations are used in this manuscript:

CoA	Coenzyme A
CDI	Carbonyldiimidazole
ECF	Ethylchloroformate
HPLC	High performance liquid chromatography
UV-Vis	Ultraviolet-visible light
