# Supplementary material for: A Chemo-Enzymatic Road Map to the Synthesis of CoA Esters
Source: Molecules. 2016 Apr 20;21(4):517. doi: 10.3390/molecules21040517 (PMC6273144; doi:10.3390/molecules21040517)
Supplement: Supplementary file 1 [file molecules-21-00517-s001.pdf]

Supplementary Materials: A Chemo-Enzymatic Road Map to the Synthesis of CoA Esters

Dominik M. Peter, Bastian Vögeli, Niña Socorro Cortina and Tobias J. Erb

S1-1: Acetyl-CoA; calculated: 810.1336, found: 810.1333.

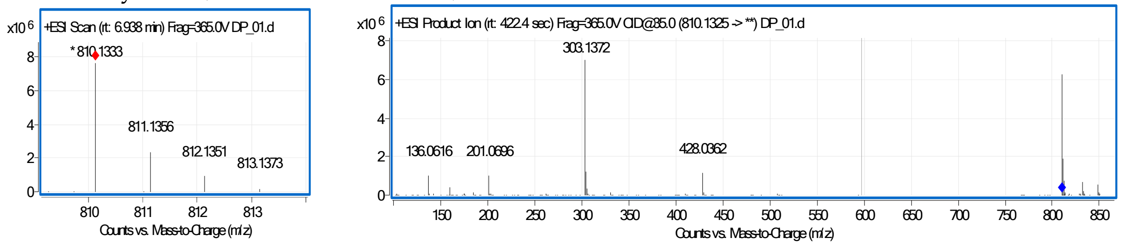

S1-2: Propionyl-CoA; calculated: 824.1493, found: 824.1493.

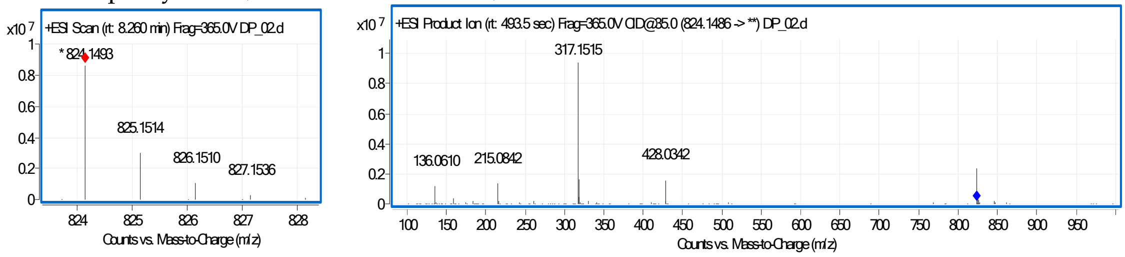

S1-3: Butyryl-CoA; calculated: 838.1649, found: 838.1633.

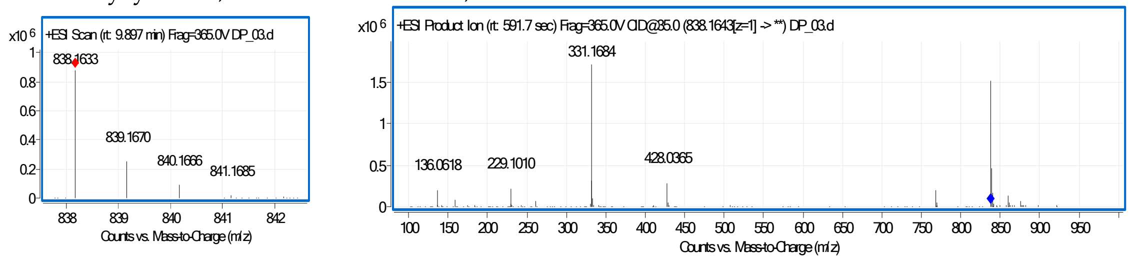

S1-4: Hexanoyl-CoA; calculated: 866.1962, found: 866.1959.

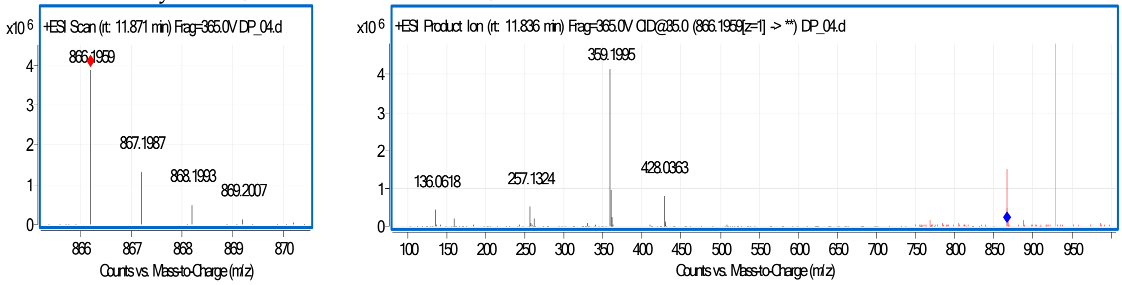

S1-5: Heptanoyl-CoA; calculated: 880.2119, found: 880.2119.

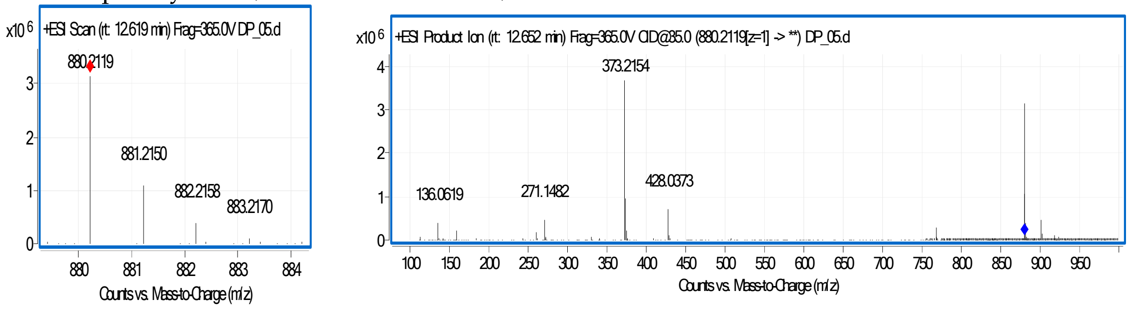

S1-6: Octanoyl-CoA; calculated: 894.2275, found: 894.2281.

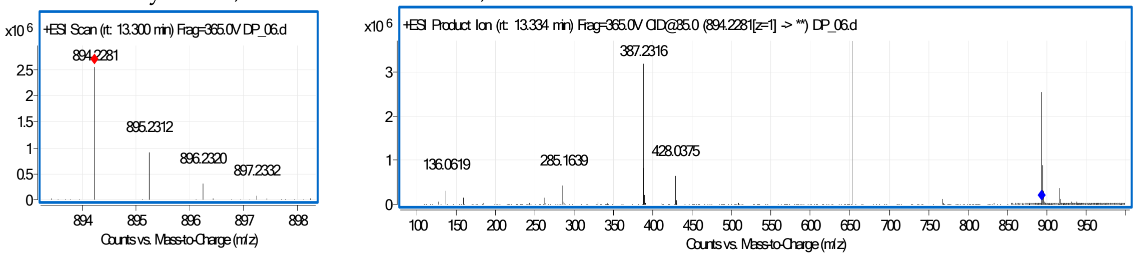

S1-7: Lauryl-CoA; calculated: 950.2901.

The corresponding mass could not be detected, presumably due to hydrophobicity of the side chain. However, a clear UV-Vis peak was detected that corresponds to the expected retention time of Lauryl-CoA

S1-8: Acrylyl-CoA; calculated: 822.1336, found: 822.1344.

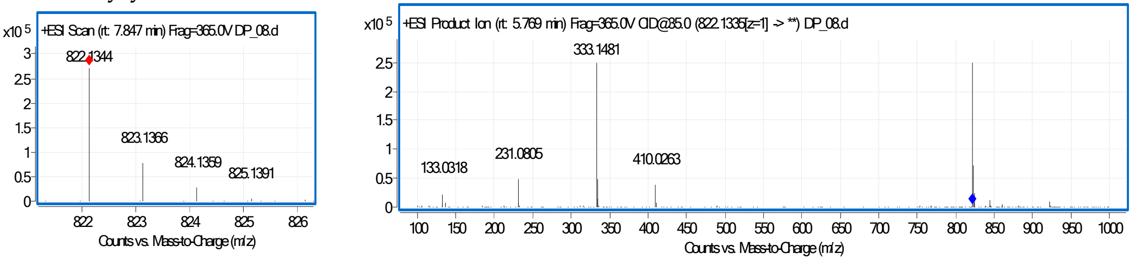

S1-9: 3,3-Dimethylacrylyl-CoA; calculated: 850.1649, found: 850.1646.

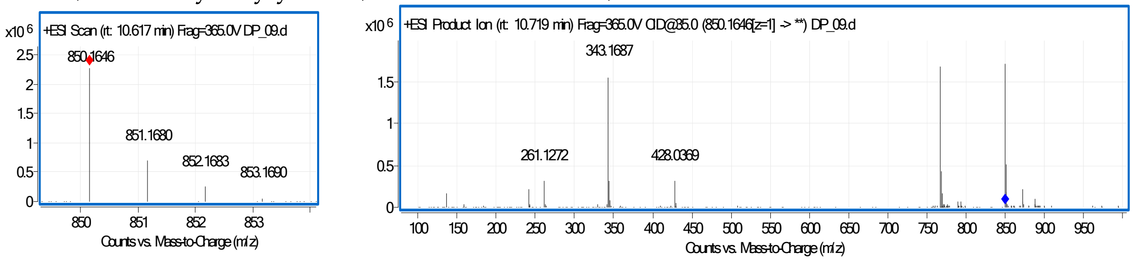

S1-10: Crotonyl-CoA; calculated: 836.1493, found: 836.1488.

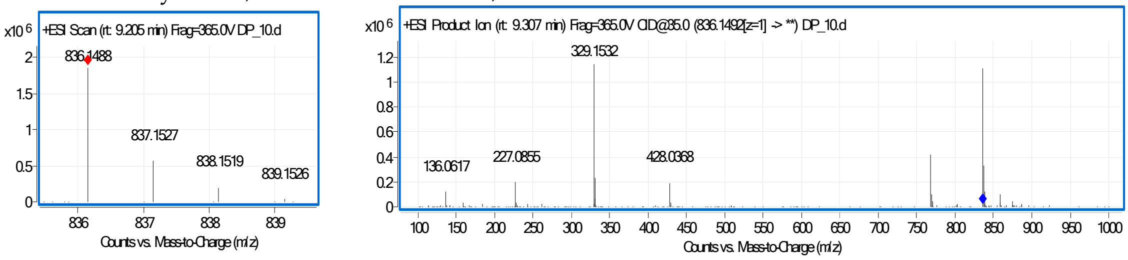

S1-11: Octenoyl-CoA; calculated: 892.2119, found: 892.2111.

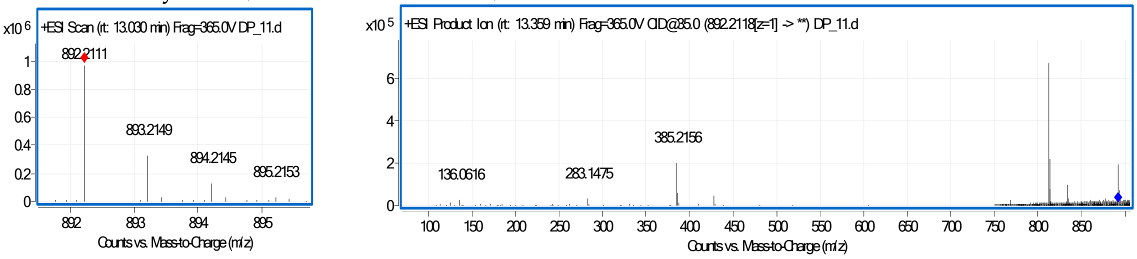

S3-12: Sorbityl-CoA; calculated: 862.1649, found: 862.1635.

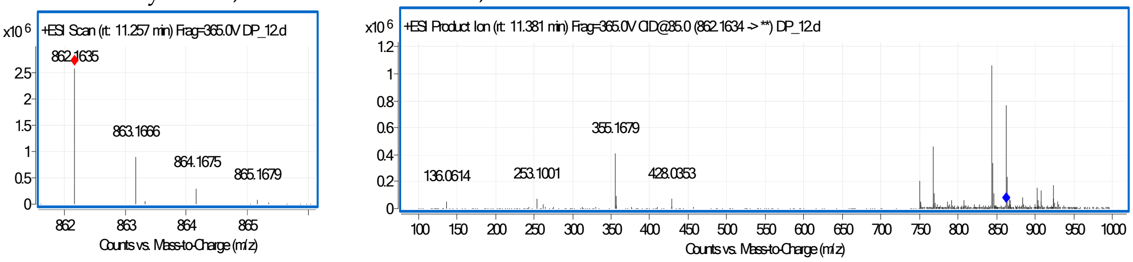

S1-13: Cinnamoyl-CoA; calculated: 898.1649, found: 898.1651.

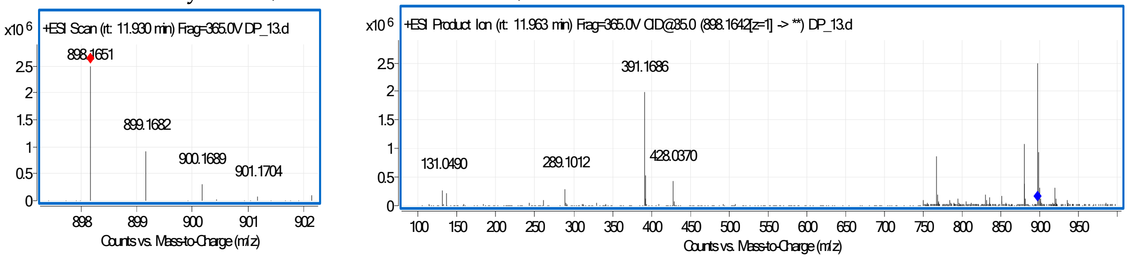

S1-14: Isobutyryl-CoA; calculated: 838.1649, found: 838.1625.

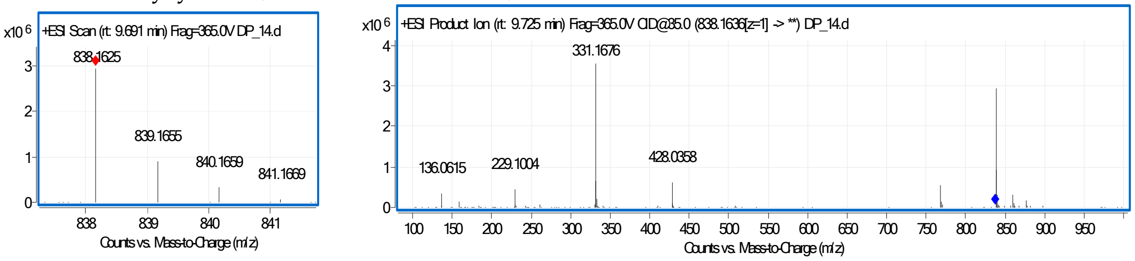

S1-15: 2-Methylbutyryl-CoA; calculated: 852.1806, found: 852.1794.

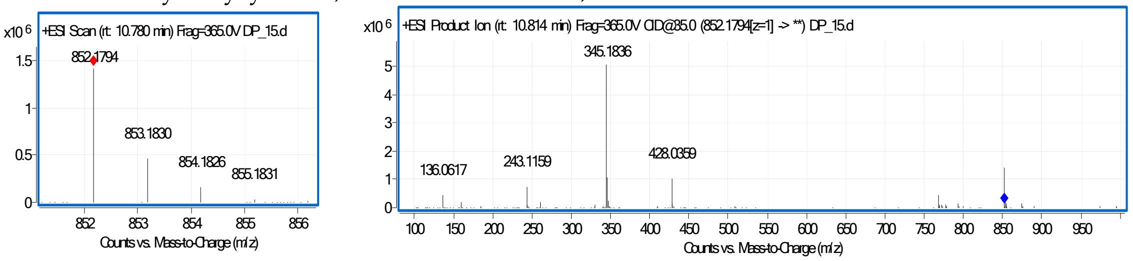

S1-16: 3-Hydroxypropionyl-CoA; calculated: 840.1442, found: 840.1442.

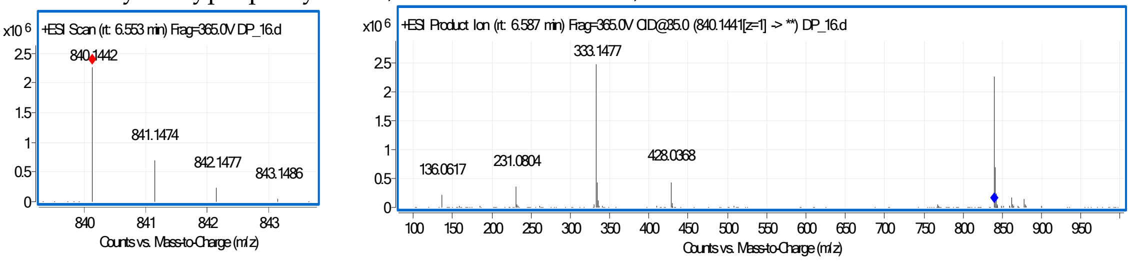

S1-17: 3-(*R*)-Hydroxybutyryl-CoA; calculated: 854.1598, found: 854.1602.

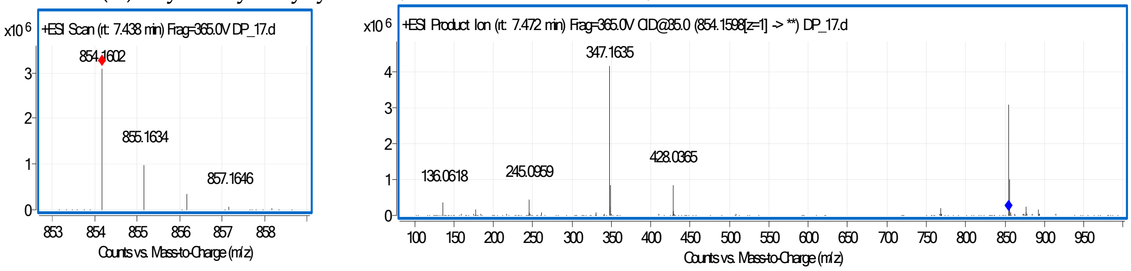

S1-18: 3-(*S*)-Hydroxybutyryl-CoA; calculated: 854.1598, found: 854.1602.

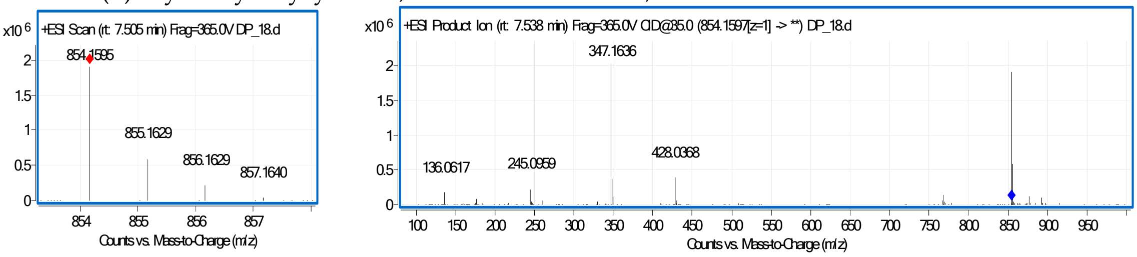

S1-19: 6-Oxoheptanoyl-CoA; calculated: 894.1911, found: 894.1907.

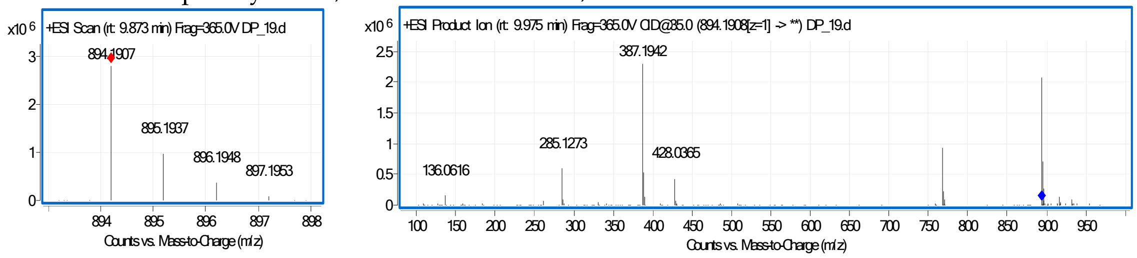

S1-20: Succinyl-CoA; calculated: 868.1391, found: 868.1387.

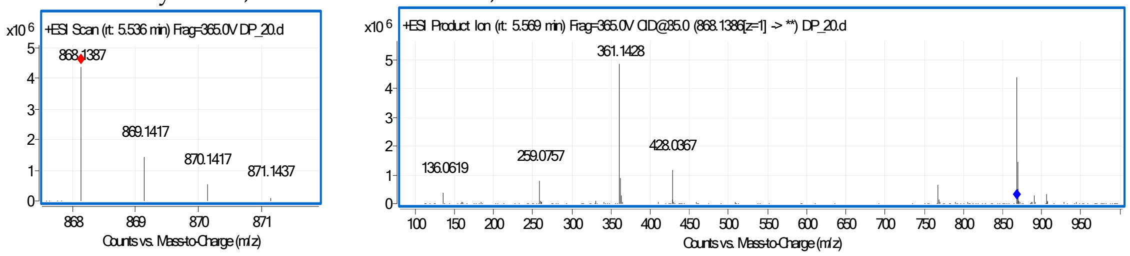

S1-21: Methylsuccinyl-CoA; calculated: 882.1547, found: 882.1541.

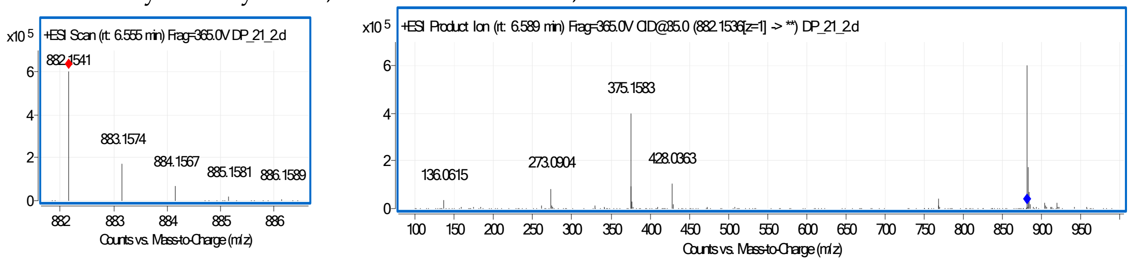

S1-22: Hexenoyl-CoA; calculated: 864.1806, found: 864.1806.

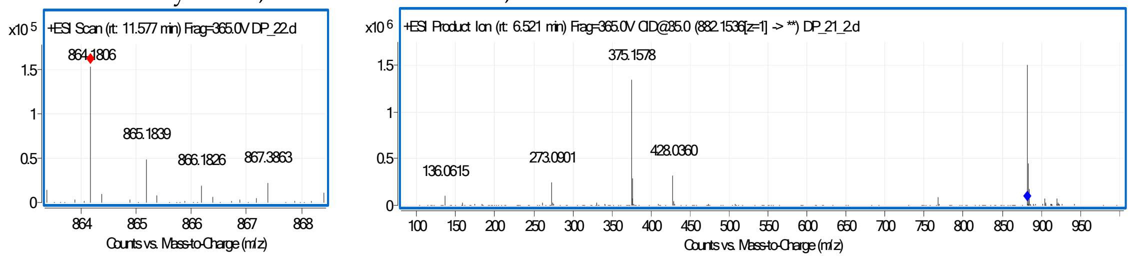

S1-23: Heptenoyl-CoA; calculated: 878.1962, found: 878.1964.

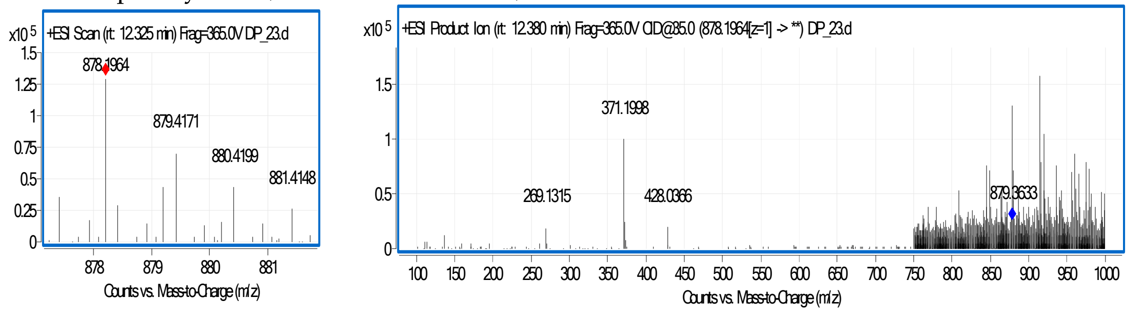

S1-24: 2-Methylacrylyl-CoA; calculated: 836.1493, found: 836.1488.

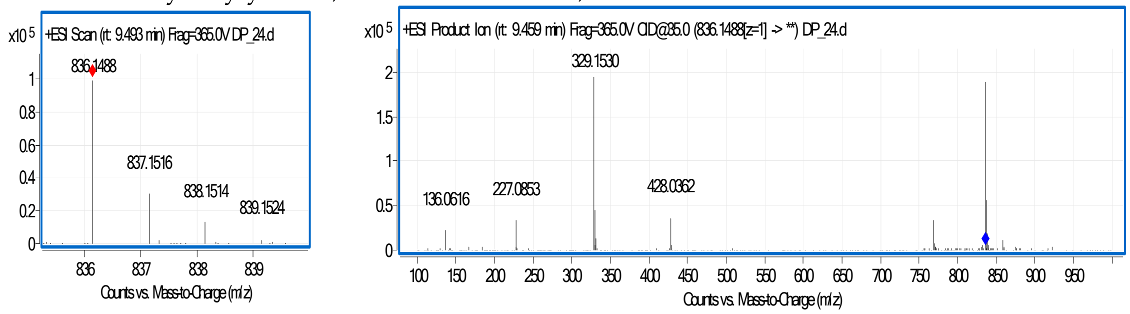

S1-25: Tiglyl-CoA; calculated: 850.1641, found: 850.1649.

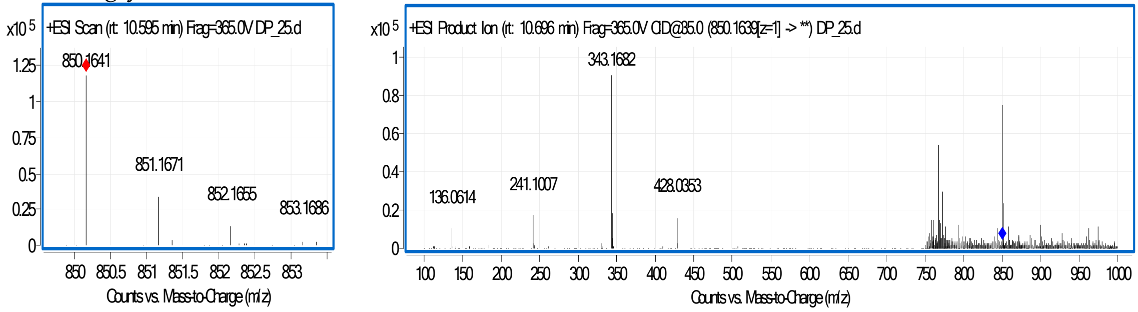

S1-26: 6-Oxoheptenoyl-CoA; calculated: 892.1755, found: 892.1739.

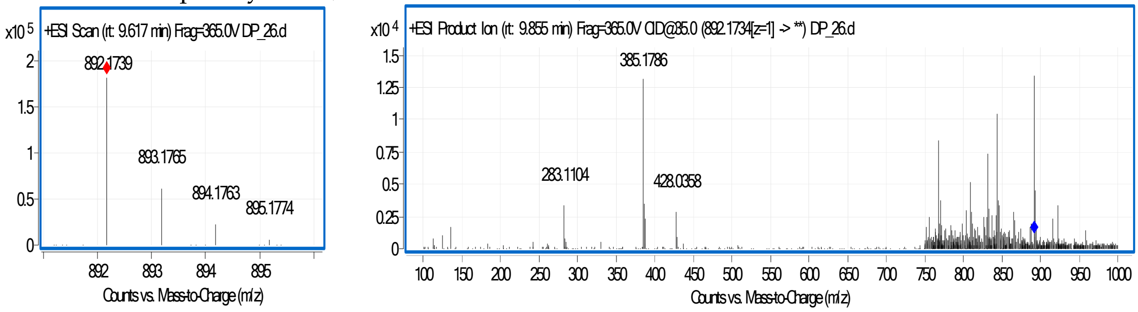

S1-27: Glyoxylyl-CoA; calculated: 824.1129.

Not synthesized in this study.

S1-28: Methylmalonyl-CoA; calculated: 868.1391, found: 868.1391.

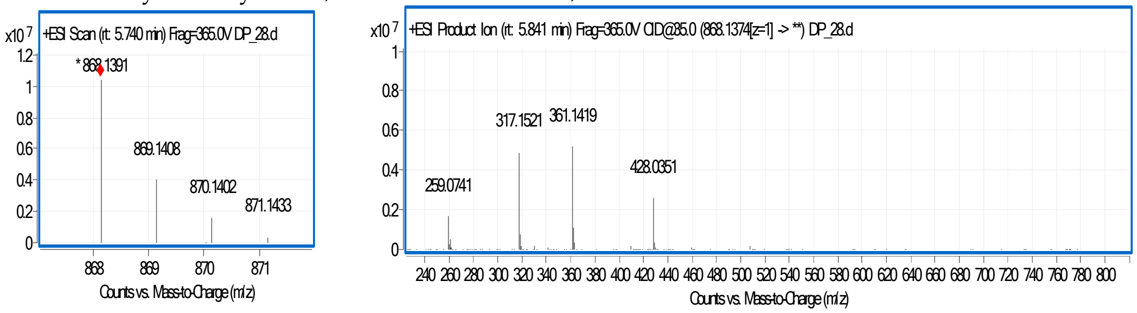

S1-29: Ethylmalonyl-CoA; calculated: 882.1547.

Not synthesized in this study.

S1-30: Malonyl-CoA; calculated: 854.1234, found: 854.1228.

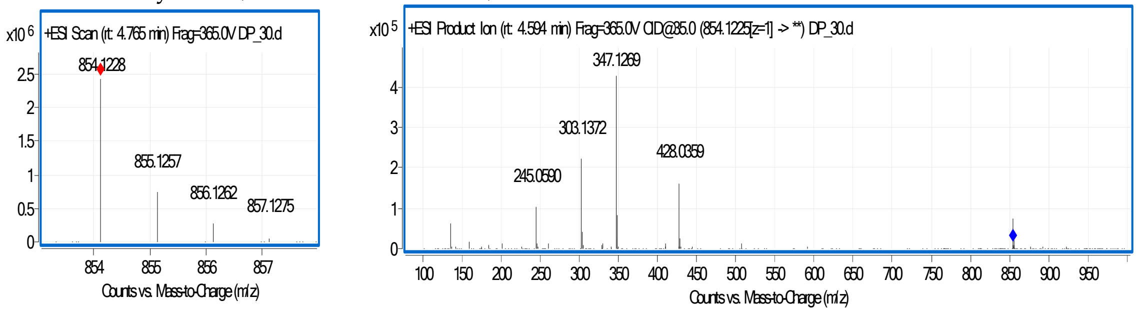

Figure S1. High resolution MS and MS/MS spectra of the CoA thioesters synthesized in this study. CoA thioesters were analyzed using an Agilent 6550 iFunnel Q-TOF LC-MS system equipped with an electrospray ionization source set to positive ionization mode. Compounds were separated on a RP-18 column (50 mm × 2.1 mm, particle size 1.7 µm, Kinetex XB-C18, Phenomenex) using a mobile phase system comprised of 50 mM ammonium formate pH 8.1 (A) and methanol (B). Chromatographic separation was carried out using the following gradient condition at a flow rate of 250 µL/min: 0 min 0% B; 1 min 0% B, 3 min 2.5% B; 9 min 23% B; 14 min 80 % B; 16 min 80%; 17 min 0% B; 18 min 0% B. Capillary voltage was set at 3.5 kV and nitrogen gas was used as nebulizing (20 psig), drying (13 L/min, 225 °C) and sheath gas (12 L/min, 400 °C). The TOF was calibrated using an ESI-L Low Concentration Tuning Mix (Agilent) before measurement (residuals less than 0.04 ppm for ten reference ions) and was recalibrated during a run using 922 *m*/*z* as reference mass. MS data were acquired with a scan range of 750–1000 *m*/*z*. The CoA esters were fragmented by collision induced dissociation with an isolation width of approximately 4 *m*/*z*, a collision energy of 35 eV, and an acquisition time of 1000 ms/spec.

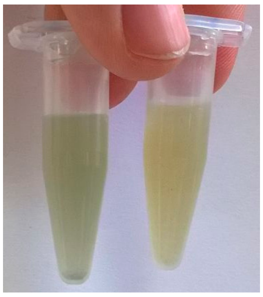

Figure S2. Examples of acyl-CoA dehydrogenase screen assays. The progress of the dehydrogenase reaction can be easily followed by eye as the reaction mixtures turns from light blue to yellow and starts to precipitate. On the left side, the almost not reacted assay of 424 with isobutyryl-CoA after 2 h of incubation. On the right side, the completely reacted assay of 605 with isobutyryl-CoA after 2 h of incubation.

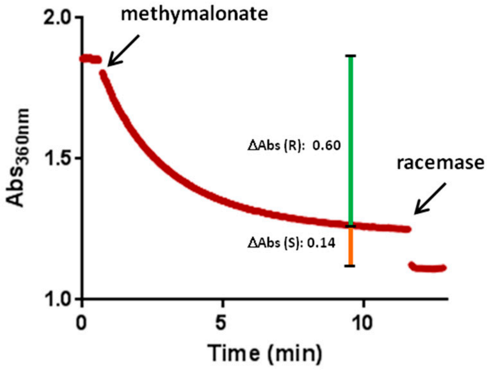

Figure S3. Spectrophotometric assay for the determination of the stereochemical outcome of the MatB catalyzed ligation of CoA and methylmalonyl-CoA. The assay containing ATP, CoA, NADPH, $MgCl_2$, NaCl MatB, Mcm, SucD in NaPO4 buffer at pH 8 was started by adding methylmalonate and the progress of the reaction was followed at 340 nm at 30 °C. After the reaction reached steady state methylmalonyl-CoA racemase was added. The ΔAbs_{360nm} before the addition of the racemase corresponds to the consumption of (*S*)-methylmalonyl-CoA produced by MatB, the ΔAbs_{360nm} after the addition to (*R*)-methylmalonyl-CoA.

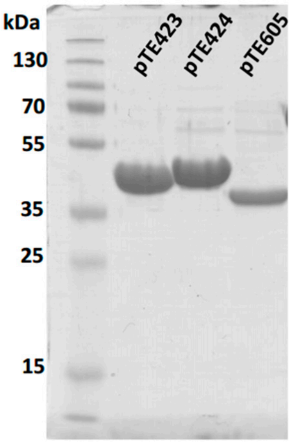

Figure S4. SDS page gel of the three acyl-CoA dehydrogenases screened. Expected size for 423: 42.5 kDa; for 424: 43.6 kDa, and for 605: 41.8 kDa. Molecular weight marker: PageRuler Plus Prestained Protein Ladder (Thermo scientific).

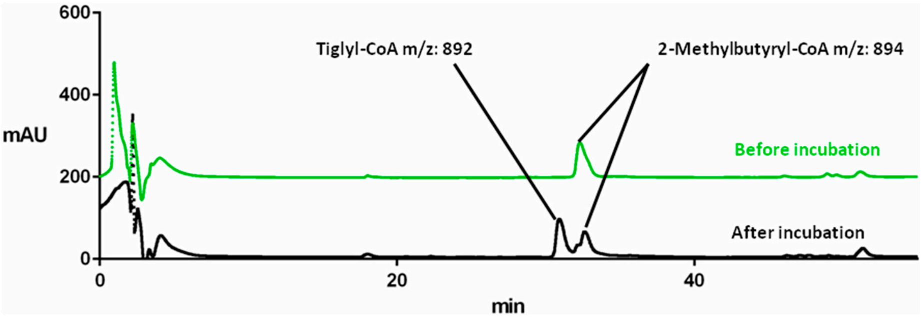

Figure S5. Model UV-Vis trace of 2-Methylbutyryl-CoA before and after desaturation by 424. Green: before desaturation reaction, black: after incubation with 424.

Table S1. Results of syntheses not listed in Table 1.

Acyl-CoA	Yield (%)	Used Method
Butyryl-CoA **3**	40	CDI
Acrylyl-CoA **8**	0	CDI
3,3-Dimethylacrylyl-CoA **9**	4	CDI
Crotonyl-CoA **10**	0	CDI
Octenoyl-CoA **11**	2	CDI
Sorbityl-CoA **12**	9	CDI
Cinnamoyl-CoA **13**	1	CDI
Methylmalonyl-CoA **28**	0	CDI
Ethylmalonyl-CoA **29**	0	CDI
Glyoxylyl-CoA **27**	0	CDI
Methylmalonyl-CoA **28**	0	ECF
Ethylmalonyl-CoA **29**	0	ECF
Glyoxylyl-CoA **27**	0	ECF
Crotonyl-CoA **10**	44	ECF

Table S2. Protein sequence of the three acyl-CoA dehydrogenases screened.

Protein	Protein Sequences
**423**	MGSSHHHHHHSSGLVPRGSHMSVLTDEQRLVQEMARTFAREVLAPGAAARAKAKAIE<br>PAVLAQMGELGFFGMTVPEEMGGVGADYMSYALALIEIAAGDGAVSTVMSVHNAPFN<br>AILQRFASPAQRERVLRPAAQGAFIGAFALTEAHAGSDASALRSRARRAGGDYVIDGEK<br>VFITSGRLAGWAILFARMEGSTGKEGITCFLTPTDTPGYEVVKVEDKLGQEASDTCALRF<br>DSLRVPEALRIGAEGEGYRIALSSLETGRIGIAAQSVGMAQAALEAAVAYARERTSFGRP<br>LIEHQAVGFRLAEAKTRLEAARQMVLHAARMKDAGQPCLTEAAMAKLFASEAAERIV<br>SDAIQTFGGYGYSRDFPVERIYRDVRVCQIYEGTSDIQKMLILRGMA
**424**	MGSSHHHHHHSSGLVPRGSHMAGSADFDLYRPSEEHDMLRDAVRSLAEAKIAPYAAA<br>VDEEARFPQEALDALTANDLHAVHVPEEYGGAGADALATVIVIEEVARACASSSLIPAV<br>NKLGSLPVILSGSEELKKKYMTPLAKGDGMFSYCLSEPDAGSDAAGMKTRAVRDGDFW<br>VLNGVKRWITNAGVSEYYTVMAVTDPDKRSKGISAFVVEKSDEGVSFGAPEKKLGIKGS<br>PTREVYLDNVRIPADRMIGAEGTGFATAMKTLDHTRITIAAQALGIAQGALDYAKGYV<br>KERKQFGKPIADFQGIQFMLADMAMKIEAARQLTYAAAAKSQRGDSDLTFQGAAAKC<br>FASDVAMEVTTDAVQLLGGYGYTRDYPVERMMRDAKITQIYEGTNQVQRIVMARNLP
**605**	MGHHHHHHHHHHSSGHIEGRHMDFALSEEQQAIFDMARAFGAEEIAPHARAWEEA<br>GTIPRTLWPKVAELGLGGVYVSEDHGGSGLGRLDATLVFEALAMACPSVAAFLSIHNM<br>CAGMIDRYGSEELKARWLPGICALTTLVSYCLTEPGAGSDAAALRTRADATPEGYRLSG<br>TKAFISGGGYSDAYLTMCRTGGAGPKGISTLLVPAGTPGLSFGGLEDKMGWRAQETRQ<br>VQFDECLVSTDLLVGEEGQGFAYAMAGLDGGRLNIAATALGGAQAAFDATRAYMAER<br>KAFGQRLDGFQALQFRLAEMEVKLQQARIFLRQAAWKLDQGAPDATKFCAMAKLAV<br>TDSAFEVANQCLQLHGGYGYLADYGIEKIVRDLRVHQILEGTNEIMRVIVARALGAA

Table S3. DNA sequence of the three acyl-CoA dehydrogenases screened.

Protein	DNA Sequences
**423**	ATGGGCAGCAGCCATCATCATCATCATCACAGCAGCGGCCTGGTGCCGCGCGGC<br>AGCCATATGAGCGTCCTGACCGACGAGCAGCGCCTCGTGCAGGAGATGGCGCGC<br>ACCTTCGCCCGCGAGGTGCTGGCACCCGGCGCCGCGGCCCGCGCGAAGGCCAAG<br>GCGATCGAGCCGGCCGTGCTCGCGCAGATGGGCGAGCTCGGGTTCTTCGGCATG<br>ACGGTGCCCGAGGAGATGGGCGGCGTCGGCGCCGACTATATGAGCTATGCGCTG<br>GCGCTGATCGAGATCGCGGCGGGCGACGGCGCGGTCTCGACGGTGATGAGCGTG<br>CATAACGCCCCCTTCAACGCGATCCTCCAGCGGTTCGCGAGCCCCGCGCAGCGC<br>GAGAGGGTGCTGCGGCCGGCGGCACAGGGCGCCTTCATCGGCGCCTTCGCCCTG<br>ACCGAGGCCCATGCGGGTTCGGACGCCTCGGCGCTGCGCAGCCGGGCGCGGCGC<br>GCGGGCGGAGACTATGTGATCGACGGCGAGAAGGTCTTCATCACCTCGGGGCGG<br>CTCGCGGGCTGGGCAATCCTGTTCGCGCGGATGGAGGGAAGCACGGGCAAGGA<br>GGGCATCACCTGCTTCCTCACCCCCACCGACACGCCGGGCTACGAGGTGGTCAA<br>GGTCGAGGACAAGCTCGGGCAGGAGGCGTCCGACACCTGCGCGCTGCGCTTCGA<br>CAGCCTGCGGGTGCCCGAGGCGCTGCGGATCGGGGCCGAGGGCGAGGGCTACC<br>GGATCGCGCTCTCCAGCCTCGAGACCGGGCGCATCGGCATCGCGGCCCAGTCGG<br>TCGGCATGGCGCAGGCGGCGCTCGAGGCGGCGGTGGCCTATGCGCGCGAGCGG<br>ACCTCGTTCGGGCGGCCGCTGATCGAGCATCAGGCGGTGGGCTTCCGGCTTGCCG<br>AGGCGAAGACGCGGCTCGAGGCGGCGCGGCAGATGGTGCTTCATGCGGCGCGG<br>ATGAAGGATGCGGGCCAGCCCTGCCTGACCGAGGCCGCGATGGCCAAGCTCTTC<br>GCCTCGGAGGCGGCCGAGCGGATCGTGTCGGACGCGATCCAGACCTTCGGCGGC<br>TACGGCTACAGCCGCGACTTTCCCGTGGAGCGGATCTACCGCGATGTCCGGGTGT<br>GCCAGATCTACGAGGGCACCTCGGACATCCAGAAGATGCTGATCCTCAGGGGCA<br>TGGCGTGA
**424**	ATGGGCAGCAGCCATCATCATCATCATCACAGCAGCGGCCTGGTGCCGCGCGGC<br>AGCCATATGGCCGGATCGGCTGACTTCGACCTGTACCGCCCGTCCGAGGAGCAC<br>GACATGCTCCGGGACGCCGTCCGCTCGCTGGCCGAGGCGAAGATCGCGCCGTAC<br>GCCGCCGCCGTGGACGAGGAGGCGCGCTTCCCGCAGGAGGCGCTGGACGCGCTC<br>ACCGCGAACGACCTGCACGCGGTGCACGTCCCCGAGGAGTACGGCGGCGCGGG<br>CGCCGACGCGCTCGCCACGGTCATCGTGATCGAGGAGGTGGCCCGCGCCTGCGC<br>GTCCTCCTCCCTCATCCCGGCCGTGAACAAGCTCGGCTCGCTCCCGGTGATCCTCT<br>CCGGCTCCGAGGAGCTGAAGAAGAAGTACATGACCCCGCTCGCCAAGGGCGAC<br>GGCATGTTCTCCTACTGCCTCTCCGAGCCCGACGCCGGCTCCGACGCGGCCGGCA<br>TGAAGACCAGGGCCGTCCGCGACGGCGACTTCTGGGTGCTCAACGGCGTCAAGC<br>GCTGGATCACCAACGCCGGCGTCAGCGAGTACTACACCGTCATGGCGGTCACCG<br>ACCCCGACAAGCGCTCCAAGGGCATCTCCGCCTTCGTGGTCGAGAAGTCCGACG<br>AGGGCGTGTCCTTCGGCGCCCCGGAGAAGAAGCTCGGCATCAAGGGCTCCCCGA<br>CCCGCGAGGTCTACCTCGACAACGTCCGCATCCCCGCCGACCGCATGATCGGCG<br>CGGAGGGCACCGGCTTCGCCACCGCGATGAAGACGCTGGACCACACCCGCATCA<br>CCATCGCCGCCCAGGCCCTCGGCATCGCCCAGGGCGCCCTCGACTACGCCAAGG<br>GCTACGTCAAGGAGCGCAAGCAGTTCGGCAAGCCGATCGCCGACTTCCAGGGCA<br>TCCAGTTCATGCTCGCCGACATGGCCATGAAGATCGAGGCCGCCCGCCAGCTGA<br>CGTACGCGGCGGCCGCCAAGTCGCAGCGCGGCGACAGCGACCTGACCTTCCAGG<br>GCGCCGCCGCCAAGTGCTTCGCCTCGGACGTGGCCATGGAGGTCACCACGGACG<br>CCGTCCAGCTGCTCGGCGGCTACGGCTACACCCGGGACTACCCGGTGGAGCGCA<br>TGATGCGCGACGCCAAGATCACGCAGATCTACGAGGGCACCAACCAGGTCCAG<br>CGCATCGTCATGGCGCGCAACCTGCCGTAG
**605**	ATGGGCCATCATCATCATCATCATCATCATCATCACAGCAGCGGCCATATCGAAG<br>GTCGTCATATGGATTTCGCGCTGAGCGAGGAGCAACAGGCGATCTTCGACATGG<br>CACGCGCGTTCGGGGCCGAGGAGATCGCCCCCCATGCGCGGGCCTGGGAAGAG<br>GCGGGGACGATCCCCCGCACGCTCTGGCCCAAGGTGGCGGAACTGGGTCTGGGC<br>GGGGTCTATGTCTCGGAAGATCATGGAGGATCCGGGCTCGGGCGGCTCGATGCG<br>ACGCTCGTCTTCGAGGCGCTGGCCATGGCCTGTCCGTCGGTTGCGGCGTTCCTCTC<br>GATCCACAACATGTGCGCGGGCATGATCGACCGCTATGGCTCGGAGGAGCTGAA<br>GGCGCGCTGGCTGCCGGGCATCTGCGCGCTCACCACGCTCGTCTCCTACTGCCTG<br>ACCGAGCCCGGAGCGGGATCGGACGCGGCGGCGCTCCGCACCCGCGCCGACGC

	CACCCCAGAAGGCTACCGGCTGAGCGGGACCAAGGCCTTCATCTCCGGAGGCGG<br>CTATTCGGACGCCTATCTCACCATGTGCCGCACCGGCGGCGCGGGGCCGAAGGG<br>CATCTCGACGCTCCTCGTGCCGGCGGGAACCCCCGGCCTCAGTTTCGGCGGGCTC<br>GAGGACAAGATGGGCTGGCGGGCGCAGGAGACGCGGCAGGTGCAGTTCGACGA<br>GTGCCTCGTGTCGACGGATCTTCTGGTCGGCGAGGAGGGGCAGGGCTTTGCCTAT<br>GCGATGGCGGGGCTCGACGGCGGACGGCTCAATATCGCGGCCACCGCTCTCGGC<br>GGCGCGCAGGCAGCCTTCGACGCGACCCGCGCCTACATGGCGGAACGGAAGGC<br>TTTCGGGCAGCGGCTCGACGGGTTTCAGGCGCTGCAGTTCCGACTGGCCGAGATG<br>GAGGTGAAGCTCCAGCAGGCGCGGATCTTCCTGCGGCAGGCGGCCTGGAAGCTC<br>GATCAGGGCGCCCCCGACGCCACCAAGTTCTGCGCCATGGCCAAACTCGCGGTG<br>ACGGATTCGGCCTTCGAGGTGGCCAACCAGTGCCTTCAGCTGCACGGGGGCTAC<br>GGCTACCTTGCCGATTACGGCATTGAGAAGATCGTGCGCGACCTGCGCGTGCAC<br>CAAATTCTCGAAGGCACCAACGAGATCATGCGGGTGATCGTGGCCCGGGCCTTG<br>GGGGCCGCATGA
